# Nano-Engineered Surface Comprising Metallic Dendrites for Biomolecular Analysis in Clinical Perspective

**DOI:** 10.3390/bios12121062

**Published:** 2022-11-22

**Authors:** Rohini Kumari, Daphika S. Dkhar, Supratim Mahapatra, Divya, Surinder P. Singh, Pranjal Chandra

**Affiliations:** 1 Laboratory of Bio-Physio Sensors and Nanobioengineering, School of Biochemical Engineering, Indian Institute of Technology (BHU), Varanasi 221005, India; 2 CSIR—National Physical Laboratory, Dr. K. S. Krishnan Marg, New Delhi 110012, India; 3 Academy of Scientific and Innovative Research (AcSIR), Ghaziabad 201002, India

**Keywords:** nanodendrites, nanoengineering, electrochemical biosensor, immunosensor, biomarkers

## Abstract

Metallic dendrites, a class of three-dimensional nanostructured materials, have drawn a lot of interests in the recent years because of their interesting hierarchical structures and distinctive features. They are a hierarchical self-assembled array of primary, secondary, and terminal branches with a plethora of pointed ends, ridges, and edges. These features provide them with larger active surface areas. Due to their enormous active areas, the catalytic activity and conductivity of these nanostructures are higher as compared to other nanomaterials; therefore, they are increasingly used in the fabrication of sensors. This review begins with the properties and various synthetic approaches of nanodendrites. The primary goal of this review is to summarize various nanodendrites-engineered biosensors for monitoring of small molecules, macromolecules, metal ions, and cells in a wide variety of real matrices. Finally, to enlighten future research, the limitations and future potential of these newly discovered materials are discussed.

## 1. Introduction

Nanomaterials have emerged as a fascinating category of materials in the past few years, with a wide range of applications [1]. In comparison to bulk materials, the electrical characteristics, size-dependent effects, and mechanical properties can be significantly changed at the nano-scale level [2]. Among several morphologies, noble metal nanostructures with branching architectures have gained much scientific attention because of their excellent catalytic performance. This unique performance of tree-like structures originates from their high porosity, large surface area, abundant edge/corner atoms, high index exposed facets, etc. [3]. For example, bimetallic platinum-on-palladium (Pt-on-Pd) nanodendrites show increased catalytic activity for oxygen reduction reactions (ORR) [4]. Furthermore, hierarchical nanodendritic structures have interconnected branches that serve as bridges between each subunit, facilitating electron transmission in a reaction [5]. Nanodendrites also impart numerous sharp ends where electrodeposition occurs preferentially, resulting in a strong electric field that improves catalytic activity by speeding up the reactant movement [6]. Dendrites are observed in crystalline or metallic elements (for example, silver (Ag), gold (Au), and copper (Cu)) and can be macromolecules, supramolecules, or nanostructures, whereas dendrimers are heavily branched supramolecules or macromolecules [7]. Metallic dendritic nanostructures are extensively employed in various domains, including solar energy storage, medicinal applications, spectroscopy, and biosensing [8]. Various monometallic nanodendrites based on nickel (Ni), Cu, Au, platinum (Pt), Ag, cobalt (Co), and other metals have been utilized for the fabrication of biosensors due to their high conductivity [9,10]. These metals have drawn significantly more attention than bismuth because agglomeration and structural issues pose significant barriers in the development of robust BiNDs [11]. Although monometallic nanodendrites are widely used in many fields such as catalysis, optics, and biomedicine, their physicochemical properties are less superior than bimetallic or trimetallic nanodendrites and several morphological topologies, including alloys, core–shell, mixed structures, and sub-clusters, etc. [12]. The interferences of these monometallic counterparts are tackled by rigorous nanoengineering of the dendritic system which includes reaction time, type of nanostructured options, and while operating the sensors in a controlled potential window and/or fixed applied potential. Furthermore, in recent years, hybrid nanostructures composed of different metals are gaining more attention due to the synergistic interactions between two or more metals. The sensitivity, selectivity, stability, biocompatibility, and catalytic activity of hybrid nanostructures are enhanced compared to monometallic nanodendrites [13]. They can be constructed by combining distinct metals, which can either form an alloy, a core shell, or a hybrid nanostructure with a core shell and alloy [14,15]. This type of integrated nanostructure will increase the sensor’s analytical performance and pave the way for the development of new nanostructures with varied dendritic nanohybrids.

Based on the scientific survey conducted through the online database “Scopus”, we found that no such review articles have been published in recent years for surface analysis based on metallic nanodendrites. In light of the enormous features and potential of these dendritic nanostructures, a new evaluation is required to assess the recent articles on the same. In this review, we have emphasized the nanodendrites-based surfaces produced by various modification processes, as well as their vast range of applications for evaluating diverse materials, namely ions, small molecules, macromolecules, and cells. Firstly, we have examined the properties and various methods of synthesis of this newly discovered material. Next, we have explained the growth models of metallic nanodendrites formation. Further, we have scrutinized the recently engineered sensing devices, fabricated with nanodendritic materials, for surface analysis with the help of various illustrative schemes. Finally, we have included an extensive tabulated form of nanodendrites-based sensors, with their sensing mechanism, description, response time, real sample, dynamic range, readout system, and limit of detection (LOD). Additionally, the limitations and future potential of these newly discovered materials were explored in order to promote further research.

## 2. Properties

Metallic nanodendrites have diverse properties owing to their high structural complexity compared to nanospheres, nanodiscs, and nanowires. They possess immense catalytic, magnetic, optical, and electronic properties [16]. For instance, Guo and his colleagues discovered that bimetallic nanodendrites Pt/Au showed enhanced optical properties than the core/shell gold@platinum nanoparticles (Au@PtNPs) [17]. This was due to the dipolar plasmonic oscillation caused by the deposition of Pt on the core, composed of Au. When light illuminates metallic nanoparticles (1–100 nm), free electrons on the particles’ surfaces get stimulated, and as a result, the electron cloud is distributed asymmetrically over the nanoparticles. A string of oscillations is generated by the movement of electrons, resulting in an exciting process known as localized surface plasmon resonance (LSPR) [18]. Similarly, metallic nanodendrites such as Cu, palladium (Pd), and Ag produce extremely confined electrical fields within the particle’s boundary and hence, localized surface plasmon resonance (LSPR), when exposed to an adequate frequency of incident light. The surface plasmon resonance (SPR) can be visualized in the first NIR (600–800 nm) or the second NIR (900–1200 nm) based on the dielectric characteristics, size, morphological alignment, and composition of the nanodendrites [19]. These factors have a significant impact on the plasmonic nanodendritic structures’ capability for scattering and absorption. For example, Huang et al. studied the absorption spectrum of Au nanodendrites floating in the water in the UV–Vis region. They found that from 500 nm to NIR, the spectrum showed a progressive rise in absorption [20]. The highly branched metallic dendrites displayed increased surface roughness and facets, which can be utilized for surface applications, including surface-enhanced Raman scattering (SERS) and catalysis [21]. Its larger surface area allows SERS sensitivity to increase multiple folds. For instance, because of the multi-level branches, corners, and edges, dendritic Ag nanostructures have greater SERS hotspots than ordinary Ag nanoparticle films [22]. These branched nanodendrites show enhanced electrocatalytic activity in various electrochemical reactions (including, oxygen reduction reactions, methanol reduction reactions, and ethanol oxidation reactions), due to their large surface area, rapid mass transfer, and superior electrical conductivity [23]. For example, amino silane-assisted production of Au dendrites showed improved electro-catalytic activity for methanol oxidation, indicating that it might be used in direct-methanol fuel cells [24]. Surface or size effects of metallic dendrites are generally responsible for the unique magnetic behavior. Li et al. demonstrated that the three-dimensional (3D) platinum dendritic structure’s stable magnetism is a result of the localization of surface electrons brought about by firmly bound oxygen molecules as well as the local magnetic moment brought about by oxygen vacancies on nearer platinum and oxygen atoms [25]. Furthermore, due to the synergistic interactions between the shell and metal core of bimetallic core–shell nanodendrites, their physicochemical properties, such as magnetic, optical, catalytic, and electronic properties, have increased in comparison to their monometallic parts [26].

## 3. Methods of Syntheses

**Figure 1:**
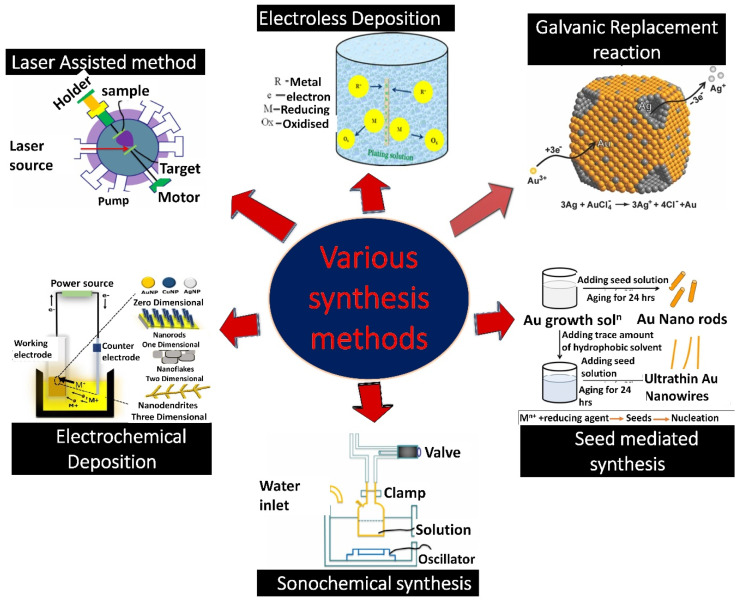
Schematic depicting the various methods of nanoparticles synthesis. Reprinted with permission from [31,32,33,34]. Copyright 2022, Tiwari et al.; Copyright 2020, Purohit et al., Copyright 2013, Xia et al., Copyright 2015, Li et al.

There are a variety of methodologies available to produce 3D nanostructured materials (NSMs) with controlled shapes and sizes. Some of them, namely galvanic replacement reactions (GRR), seed-mediated, co-reduction, sonochemical reductions, laser-assisted synthesis, electroless deposition, and electrochemical deposition, are described in this section (Figure 1). The fundamental chemical reactions involved in all nanodendrites synthetic methods are reduction. However, for a better understanding of reactions, we have discussed specific examples in the subsections below. Nanodendrites’ morphology can be controlled by utilizing reducing agents, capping agents, and ultrasonic waves [27,28]. They control the metal atoms’ migration and the subsequent deposition. The capping agent also reduces the interfacial free energy of some facets and makes them thermodynamically more desirable for the regulated growth of nanodendrites [29]. In addition, morphology can also be controlled by varying electrodeposition parameters including, concentration and volume of the electrolyte solution, electrodeposition time, and the electrodeposition current density [30].

### 3.1. Galvanic Replacement Reaction

GRR has evolved as a significant method to create highly ordered anisotropic nanodendrites to be used in biomedical field, plasmonics, and catalysis [35]. This method provides a wide range of opportunities to fabricate porous nanostructures. Luigi Galvani, an Italian physician who invented the first galvanic cell, coined the term galvanic [36]. The mechanism of this process is the replacement reaction, wherein metals of interest are replaced by first metal (sacrificial template) because of their varied reduction potential [37]. The sacrificial template gets oxidized and dissolved into the solution, and the ions of the second metal get reduced and deposited on the outer surface of the template. In GRR, the morphology of the sacrificial template plays a major role in controlling the shape of nanostructures because nanostructures get deposited on it [33]. Such chemical reactions have been utilized to develop nanodendrites in various studies. In one such case, the formation of 200 nm crystalline dendritic silver nanostructures has been formed by GRR. In this study, a Cu mesh substrate was used as a sacrificial template and replaced silver ions (Ag+) from a silver nitrate (AgNO_3_) solution. Hence, the reduction of silver ions resulted in the formation of silver nanodendrites [38,39]. Usually, reduction reactions occur on one type of template facet, however, galvanic replacement can continue with facet selectivity when multiple types of facets are there on a template’s surface. The materials created with this technique have a high porosity and a large surface area to volume ratio. However, in this technique, even minor variations in temperature and ion concentrations can affect the reduction potential’s actual value and hence reaction kinetics [40]. These variations might cause a replacement reaction to go in the opposite direction and stop or prohibit galvanic replacement. The final product’s morphology is significantly influenced by the GRR site selection on the initial template. The deposition of new atoms must take place on the low-energy facets, whereas the oxidation/dissolution of the template should occur predominantly on the surfaces with the highest surface free energy [33]. Consequently, additional studies are still required to properly understand and precisely manage the process of reduction on the facet. 

### 3.2. Seed-Mediated

The seed-mediated growth process, one of the versatile approaches, is utilized for customizing dendritic architectures of metals [41]. Murphy, in 2001, first coined the term seed-mediated growth method while preparing nanorods of Au by utilizing Au nanoparticles as seeds [42]. Many exciting studies have been published focusing on the seed-mediated synthesis of monometallic nanodendrites such as Au, Pd, Pt, etc., having distinct branches on their surface. The shape-directing substance utilized during the synthesis has been shown to significantly impact the creation of metallic nanodendrites. This method is also widely used to make bimetallic anisotropic nanodendrites. The production is mostly accomplished by a two-step process involving metal precursors. In the first step, desired seeds are synthesized. While in the second step, proliferation of the second metal occurs on the first metal’s seeds surface in the presence of reducing agent and surfactant [43]. For example, Kobayashi et al. have synthesized Pd–Rh bimetallic nanodendrites using the seed-mediated method. Firstly, in this experiment, palladium nanocrystals with truncated octahedral shapes were synthesized as seeds. Further in the second step, in the presence of polyvinylpyrrolidone (surfactant), L-ascorbic acid (reducing agent), and Na_3_RhCl_6_, Pd–Rh bimetallic nanodendrites were formed [44]. This approach has two different growth patterns: non-conformal epitaxial growth and conformal epitaxial growth [45,46]. Several parameters influence the growth process, including the rate of metal ion reduction, crystallinity plane, stabilizing agents, metal bond energy, and surface free energy [37]. However, it is not employed for the large-scale synthesis of nanomaterials because if we raise the concentration and temperature of the seeds, the aspect ratio of the nanomaterials falls and they have lesser stabilization [47].

### 3.3. Co-Reduction

The co-reduction method is believed to be the simplest and most basic method for fabricating bimetals with alloyed or intermetallic nanostructures. The simultaneous reduction of two precursor metal ions with a reducing agent, followed by the nucleation and development of nanostructures, is a key reaction in this process [37]. Various studies have used the co-reduction process to create nanodendrites. In one study, Ortiz et al. have synthesized Pd–Pt bimetallic nanodendrites using oleylamine as a reducing agent. In order to achieve co-reduction of both metals, Pd(acac)_2_ and Pt(acac)_2_ were dissolved in oleylamine at a 1:1 mol ratio and heated at 160 °C [48]. In co-reduction, both surfaces can be capped with the help of surfactants in order to regulate the shape and size of the nanostructures [49]. The rate of reduction and the ionic interactions between both metals substantially impacts the synthesis of bimetallic nanodendrites by this method. The reduction rate is directly related to the metal ion reduction potential; the higher the reduction potential, the more easily the ions are reduced. However, metals having similar reduction potentials are more likely to form alloys [50]. He et al. recently emphasized the dendritic Au–Pt bimetallic system’s fabrication process using ascorbic acid as the reducing agent. In this reaction, by altering the molar ratio of the Au^3+^/Pt^2+^ precursor ions, porous Au–Pt nanodendrites with a tunable composition were formed [19]. The co-reduction process has simple and straightforward steps, although contaminants were generated during this process [12].

### 3.4. Sonochemical Reduction

Sonochemical reduction is one of the physical techniques for generating various types of NSMs under the influence of high-intensity ultrasonic waves [51]. In this technique, acoustic cavitation plays the most important role in regulating the efficiency of the reduction reaction. Acoustic cavitation is the process in which bubbles form, grow, and implosively collapse in the liquid medium under the influence of ultrasonic waves [38]. This collapse of bubbles forms localized hot spots with a pressure of 1000 atm, a temperature of around 5000 K, and a cooling rate of over 109 K/s [52]. The thermodynamic far-from-equilibrium growth of metallic nanodendrites is facilitated by these particular conditions. Ultrasound has long been recognized as a valuable tool for shape-controlled nanostructure creation. The most notable benefit of employing ultrasound during nanostructure production is that it accelerates mass transport and reaction speed. Wang et al. have synthesized a silver dendritic nanostructure based on the approach discussed above. In this process, sonication of an aqueous solution of silver nitrate occurs in the presence of an isopropanol (reducing agent) and polyethylene glycol 400 (dispersant). The reaction time has a significant impact on the morphology of the nanostructures. Initially, only silver spheroidal nanoparticles were synthesized; however, as the ultrasonic irradiation time was increased, the silver nanoparticles began to aggregate and form silver dendrites [52]. In another study, Xiao et al. have synthesized silver dendritic architectures by utilizing the Raney nickel template and ultrasonic waves [53]. Despite its widespread usage in the synthesis and modification of nanomaterials, there are still issues to be resolved regarding the precise effect of sonication on reaction kinetics and the feasibility of industrial-scale sonoproduction of nanomaterials for bulk synthesis [54]. To examine the impacts of sonication on reaction kinetics, further quantitative study at the molecular level is required.

### 3.5. Laser-Assisted Method

The laser-assisted method has evolved as a versatile method for fabricating a wide range of nanomaterials [55]. This is another approach for regulating the metallic nanodendrites’ sizes and surface topologies. The laser-assisted method entails focusing a large amount of energy through a focus lens on a target at a specific location in order to generate surface atoms to be deposited [56]. The use of laser light to fabricate bimetallic nanodendrites with multifunctional systems has generated curiosity among researchers [57]. In one study, a laser-driven photochemical method was used to create dense and hierarchical Ag@Au nanodendrites, having an optically adjustable SPR phenomenon. The nucleation of dendritic-shaped nanoparticles is caused by continuous laser irradiation at a 532 nm wavelength on Au metals, immersed in a liquid solution of Ag. During laser ablation, rapid boiling and evaporation of the element Au induces the generation of an explosive Au plasma. Furthermore, the plasma starts to rapidly condense, and during condensation, Au nucleation occurred. The nucleation process stopped when the plasma vapor gets exhausted. Following 532 nm pulse laser irradiation and nucleation, Au nanoparticles’ SPR gets activated in order to induce the plasmon-mediated development of Ag species on the precursor by reducing Ag ions. As a result, ultra-small Ag@Au nanoparticles surface served as a substrate for the synthesis of highly branched and anisotropic Ag@Au nanodendrites by a laser-assisted method [58]. This method achieves the synthesis of highly pure nanodendrites without the use of hazardous chemicals, however its production rates are minimal [59].

### 3.6. Electroless Deposition

Electroless plating, often known as a non-galvanic deposition process, or cementation process, is a cost-effective and non-hazardous micro-electrochemical redox reaction [60]. The reaction involves the utilization of a reducing agent which provides the electrons needed to reduce the metal ions present in the electrolytic solution. Unlike electrodeposition, this process does not require an external power source for deposition [31]. For instance, Qiu et al. reported the formation of Ag dendrites on a silicon (Si) wafer by electroless metal deposition using an aqueous solution of hydrogen fluoride (HF) and AgNO_3_. In this method, silicon etching and silver deposition occurred simultaneously on the Si wafer surface. Silver atoms were first deposited as nuclei and then as three-dimensional nanodendrites on the surface of the silicon wafer. As silver was deposited, the surrounding silicon, which served as the anodes, was successfully etched away [61,62]. Unfortunately, this method has some drawbacks, including slow metal ion diffusion as well as slow deposition kinetics at an ambient temperature. Additionally, there are challenges with the film’s adherence and purity, surface selectivity, and ability to control deposit morphology [63]. 

### 3.7. Electrochemical Deposition

Electrochemical deposition is a robust, quick, and cost-effective method for the fabrication of nanomaterials. Electrodeposition, in contrast to other nanostructure production techniques, has a greater growth rate, utilizes less raw materials and energy, produces no unintended by-products, and has the ability to overcome shape constraints [64,65]. Additionally, nanoproduction requires no post-deposition processing, generates coatings on a variety of substrates, and is free of impurities [65]. In this type of deposition, nanomaterials get deposited on the electrode surface when optimum potential is applied. Positively charged metal ions from the electrolyte solutions flow towards the negatively charged cathode and are firmly bound to it, causing the selective deposition to only occur on the working electrode (cathode) [32,66]. The electrons needed for reduction and further deposition of metals are supplied by the external power source, resulting in the formation of a metal sheet film on the electrode [67]. This method has an added advantage as it minimizes the errors in current signal, produced by inappropriate material transfer on the surface of electrodes [32]. Further, the deposition of a modifier film onto the electrode improves adhesion. To obtain a uniform layer of these deposited nanomaterials, electrochemical techniques such as chronoamperometry, linear sweep voltammetry (LSV), double pulse deposition, cyclic voltammetry (CV), and sonoelectrodeposition are commonly utilized. The electrolyte solution’s pH affects deposition, which in turn influences the nanostructure uniformity on the electrode surface. Additionally, electrodeposition is a simple way to control alloy composition to generate novel multi-layers of nanodendrites that are difficult to achieve using other techniques [67]. Purohit et al. found that the co-deposition of metallic nanodendrites over the GCE electrode increased the electrocatalytic activity. In this study, charged metal ions (Au and Cu) from an electrolyte solution move towards the electrode surface (cathode) at a potential of −0.6 V. Further, reduction and deposition of metal ions occurred, and 3D nanodendritic patterns were formed on the surface of electrodes at 600 s. Thus, by tuning the optimum potential and deposition time, these three-dimensional morphologies can be easily formed on the surface and have a reduced fabrication time. The electrode detected acetaminophen at a 100–1000 nM concentration range with a LOD of 7.5 nM [68]. Some nanostructured composites require various templates, for example, Fu et al. have used an electrolyte of silver−ammonia [Ag(NH_3_)_2_OH] and a low fraction of hexadecyl trimethyl ammonium chloride (CTAC) to generate Ag dendrites/AgCl hybrid thin films on an indium tin oxide (ITO) electrode [69]. In light of the significant advantages of this method over other conventional methods, we mainly concentrated on electrodeposited nanodendrites for cell, macromolecule, and small molecule detection in this manuscript. 

## 4. Models of Nanodendrites Formation

For the growth of dendritic structures, several models of growth of metallic nanodendrites have previously been postulated, namely: (i) diffusion-limited-aggregation (DLA) model, (ii) cluster–cluster aggregation (CCA) model, and (iii) oriented attachment model [70,71]. However, oriented attachment and the DLA model are widely used for the construction of metallic dendrites under non-equilibrium conditions. In 1981, Witten and Sander introduced DLA to describe the heterogeneous growth of metallic dendrites constrained by diffusion [72,73]. In a diffusion-limited aggregation, the seed particle occupies the center of a lattice and a site far from the lattice is selected from where random walk is initiated [74]. The metal ions from an electrolytic solution move randomly towards the nucleation sites due to Brownian motion, stop, undergo reduction, and get absorbed on the surface of the fractal tree. However, if the random walker is far from the expanding cluster, it gets terminated, and a new random walk is started towards the surface. This process is repeatedly carried out to simulate the cluster growth process. The efficiency of DLA models can be significantly increased by permitting the random walkers to take long steps when they are distant from the cluster [72,75]. As diffusion increases, dendritic structures expand, and the angles between branches and trunks increase. After nucleation, aggregation occurs, resulting in the creation of primary, secondary, and tertiary branches. The surfactant or template controls the nuclear and directional aggregates, resulting in a non-equilibrium system that favors the formation of metallic nanodendrites [38]. The viscosity of the solvent influences the diffusional mass transfer of precursor molecules. Meakin and Kolbon proposed the CCA model, in this model large number of individual atoms randomly diffuse and adhere to one another forming metal clusters. Further, these clusters continue to diffuse randomly and touch other particles or clusters to produce a fractal morphological pattern [76,77]. The diffusive trajectory’s fractal dimension determines the cluster’s fractal dimension. With decrease in fractal dimension of diffusive trajectory, the fractal dimension of cluster increases [78]. The CCA models are classified into two types, the diffusion-limited cluster aggregation (DLCA) model and the reaction-limited cluster aggregation (RLCA) model, depending on the probability of aggregation following collision. The sticking probability can be used to represent the probability of aggregation. In the DLCA model, two particles stick once they collide with each other and have a sticking probability of one, resulting in faster aggregation. In the RLCA model, two particles do not stick immediately following collisions and have a sticking probability of less than one, resulting in slower aggregation [79]. In the DLA model, nanodendrites grow one particle at a time; however, in the CCA model, clusters of precursor molecules aggregate at the surface. Penn et al. in 1998 gave an important growth model called oriented attachment (OA) model. It is also called oriented aggregation or epitaxial assembly. In the oriented attachment model, smaller nanostructures collide and form loosely bound aggregates composed of randomly oriented nanostructures. These nanostructures further self-assemble along similar crystallographic orientations through their specific facets to generate larger anisotropic nanostructures at a planar interface [80,81,82]. These facets’ attachment lowers the nanostructures’ overall free energy by reducing the nanomaterials/liquid interface area. Both charge-stabilized nanomaterials in polar solvents and ligand-capped nanomaterial suspensions in nonpolar solvents are capable of undergoing oriented attachment growth [83].

## 5. Small-Molecule Detection Using Metallic Nanodendrite-Based Sensors 

Small molecules are organic molecules with low molecular weights (less than 900 Daltons) [84]. Monosaccharides, lipids, secondary messengers, various metabolites, xenobiotics, and medicines mainly come under this category. The recognition of these small molecules is crucial for various applications, including food and environmental analyses, clinical diagnostics, physiological function studies, and drug development [85]. Although there is a widespread use of spectroscopic and chromatographic techniques for small-molecule characterizations, sample clean-up, such as solid-phase extraction, is frequently required [86]. Sample preparation processes are laborious and require laboratory-based devices, costly, and time-consuming, limiting their utility [87,88,89].

Furthermore, the sensitivity of these techniques is very low to obtain the desired results [90]. Hence, fast and sensitive detection methods are needed, which can be transported easily. Biosensors are bioanalytical devices that can detect an ultralow concentration of biomarkers via optical, thermal, or electrical signals [91,92]. The biosensors are made up of three basic elements, i.e., a biorecognition element (BRE), a transducer, and an amplifier and processor [93]. The BRE identifies the target, and the transducer changes the biological recognition event into a quantifiable signal. After that, the signal is processed by a processor and amplified further to obtain a signal output. BREs or bio-receptors can be proteins, enzymes, deoxyribonucleic acid (DNA), ribonucleic acid (RNA), aptamers, and other biological entities [94]. Additionally the incorporation of novel nanostructured materials further paved a path for improved sensing of analytes [95,96]. This section discusses the engineering of nanobiosensors based on metallic nanodendrites to sense different small molecules. 

**Figure 2:**
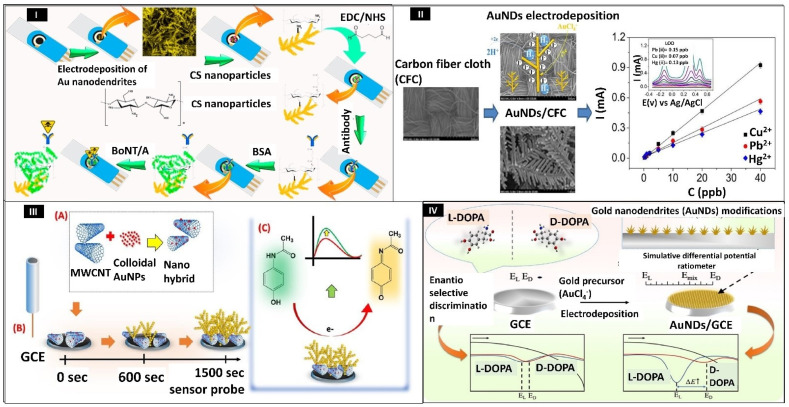
(**I**) Schematic illustration showing the immunosensing of botulinum neurotoxin type A. Reprinted with permission from [97]. Copyright 2017, Sorouri et al. (**II**) Illustration depicting carbon fiber cloth-based gold nanodendrites sensor for Pb (II), Hg (II), and Cu (II). Reprinted with permission from [98]. Copyright 2018, Dang et al. (**III**) Schematic representation of fabrication of sensor surface for detecting acetaminophen. (**A**) nanohybrid preparation, (**B**) dendrite deposition, and (**C**) AP sensing. Reprinted with permission from [9]. Copyright 2019, Purohit et al. (**IV**) Schematic illustrating the ratiometric sensing of DOPA by using gold nanodendrites. Reprinted with permission from [99]. Copyright 2020, Lian et al.

Botulinum neurotoxin (BoNT) is a harmful small molecule that poses a biological threat to humans. It is lethal at low concentrations of 1 ng/kg and thereby kept in category A of the Centers for Disease Control and Prevention’s (CDC’s) selected agents list. Among all serotypes of BoNT, BoNT/A is considered one of the most important bioweapons due to its highly toxic nature. Sorouri et al. in 2017 [97] constructed a screen-printed carbon electrode (SPCE)-based impedimetric immunosensor in order to sense BoNT/A quickly and accurately (Figure 2I). First, by using the square-wave voltammetry (SWV) approach, gold nanodendrites (AuNDs) were electrochemically coated on the surface of the SPCE to form AuNDs/SPCE. Further chitosan nanoparticles (CSNPs) were self-assembled on the surface to form unique nanocomposites of SPCE/AuNDs/CSNPs. AuNDs were successfully deposited by optimizing the potentials between −0.8 and 0.2 V. The change in the RCT was linear to the concentrations of BoNT/A in the 0.2 to 230 pg/mL range. The LOD calculated for the constructed immunosensor was estimated to be 0.15 pg/mL. The analytical performance of this device was detected in serum and milk samples [97]. Heavy metal ions, namely, mercury Hg (II), lead Pb (II), and Cu (II), are very hazardous and cause a variety of health issues such as brain function loss and Wilson’s disease. As a result, determining the amounts of such metal ions in water is crucial for evaluating human health and the environment. Dang et al. in 2018 have designed a carbon fiber cloth (CFC)-based Au nanodendrite (AuND@CFC) sensor that can simultaneously detect concentrations of Cu (II), Hg (II), and Pb (II) in water samples (Figure 2II). A piece of CFC (636 mm, 0.72 cm^2^) was cleaned first in an ultrasonic bath, followed by activation at a potential of 1.0 V for 300 s. Further, the activated CFC was electrodeposited with gold nanodendrites in 5 min. The constructed sensor has a large electroactive area owing to the nanodendrites’ hierarchical architecture. Hg (II), Pb (II), and Cu (II) were measured simultaneously in the real matrix using DPASV (differential pulse anodic stripping voltammetry). The LOD of Hg (II), Pb (II), and Cu (II) were estimated as 0.15, 0.07, and 0.13 ppb, respectively [98]. Similarly, cadmium metal also has detrimental effects on the human body which includes inflammatory reactions in the liver, respiratory system, and kidneys. Campos et al. in 2019 have designed a flexible sensor based on bismuth nanodendrites for onsite detection of cadmium and lead in sweat. In this experiment, bismuth micro/nanodendrites were electrodeposited on copper substrate and fabricated on a flexible substrate polyethylene terephthalate. The flexible copper substrate was designed using cost-effective materials, namely, an adhesive label containing the design of a three-electrode electrochemical system, flexible and adhesive conductive copper tape, and nail polish or spray as a protective layer. The calibration plot showed linearity in the range of 2–50 µM for both lead and cadmium. The detection limits of cadmium and lead were estimated to be 5.36 µM and 0.76 µM, respectively. The flexible prototypes developed have tremendous potential to be employed as perspiration-based wearable medical devices or as portable sensors to detect toxic metals [66]. Acetaminophen (AP) is generally safe for therapeutic usage, but overdoses have been linked to several negative consequences, including hepatotoxicity. In the United States, AP overdosing is responsible for 46% of all acute liver failure cases. Purohit et al. in 2019 developed novel gold nanodendrites (AuND) and multi-walled carbon nanotube gold nanoparticles (MWCNT-AuNPs) decorated surface for analysis of AP as shown in Figure 2III. First, MWCNT was sonicated for 5 min with a colloidal gold solution (1:1) to form a homogenous suspension. Then MWCNT-AuNPs composites were coated on a glassy carbon electrode (GCE) surface. Further AuND was electrochemically deposited on it using chronoamperometry at −0.30 V potential for 1500 s. The final sensor probe designed was GCE/MWCNT-AuNPs/AuND. CV, LSV, and electrochemical impedance spectroscopy (EIS) were performed for the electrochemical study. The analytical performance was carried out by LSV and differential pulse voltammetry (DPV), and based on the DPV signals, the linear range was estimated between 100 and 7500 nM. The LOD obtained was 2.12 nM. The creation of the sensor probe took a small amount of time and it can be utilized for label-free, cost-effective, and rapid sensing [9]. Neurological disorders, including Parkinson’s, Alzheimer’s, Epilepsy, and Meningitis, are the second leading cause of death worldwide. Chiral medications, which play an important function in treating symptomatic neurological illnesses, account for the majority of commercial pharmaceuticals. 3,4-Dihydroxyphenyl-L-alanine (L-DOPA), a chiral medication and precursor of the catecholamine dopamine (DA), can effectively pass the blood–brain barrier and generate DAs via metabolic processes, which may have a therapeutic efficacy on Parkinson’s disease. As a result, discrimination between isomers of a chiral drug is critical for the safety of drugs, particularly for patients with persistent neurological illnesses who require long-term therapy. Lian et al. in 2019 synthesized a robust, and extremely sensitive sensor surface for the enantio-selective sensing of 3,4-dihydroxyphenylalanine (DOPA) (Figure 2IV). In this work, AuND was electrochemically deposited on GCE by chloroauric acid (HAuCl4) solution. The AuND/GCE electrode characterizations were carried out by CVs and EIS techniques. Further, analytical performance was conducted by highly sensitive DPVs to detect the chiral molecules on AuND/GCE. The calibration plot showed the linear relationship in concentration between 10 μM and 100 μM. The biosensor developed has a lot of potential in pharmacological and pathological research involving chiral drug discrimination [99].

While chloramphenicol (CAP) is frequently utilized to treat numerous infectious illnesses due to its lower cost and good efficiency, it also represents a danger to human health. Many countries have banned CAP because of its carcinogenic effects and other severe effects, including aplastic anemia, bone marrow suppression, and serious blood diseases. However, because of the low cost and high antibacterial resistance, CAP is still widely being used. Overuse of CAP causes it to remain in the environment or groundwater. According to previous findings, it has been identified in water samples in Germany, China, and Switzerland and affects the human body. Peng et al. in 2021 [100] devised an electrochemical sensor using carbon nanotubes and copper nanodendrites. First, carboxylated MWCNTs were coated on the GCE surface, and then copper nanodendrites (CuNDs) were electrodeposited to form CuNDs/MWCNTs/GCE. The impact of deposition potential on CAP determination was investigated using CV. The maximum current of CAP was enhanced when the accumulation potential was elevated from −0.35 to −0.50 V. However, the current began to decrease progressively when they applied more than −0.50 V potential. As a result, the best deposition potential was estimated to be 0.50 V. Similarly, the best deposition time was calculated as 6 s. The electrochemical behavior of GCE, MWCNTs/GCE, CuNDs/GCE, and CuNDs/MWCNTs/GCE was studied by utilizing the EIS technique. The analytical performance of CAP was studied by CV and LSV. The dynamic range of the sensor was 0.15–12 μmol/L, and LOD was estimated as 9.84 nmol/L. Compared to all other electrode surfaces, CuNDs/MWCNTs/GCE had a larger electrocatalytic active surface and a faster electron transfer rate for the reduction of CAP. The electrocatalytic area and electron transfer rate for reduction of CAP were enhanced for CuNDs/MWCNTs/GCE compared to electrodes simply modified by MWCNTs or copper nanoparticles [100]. 

**Figure 3:**
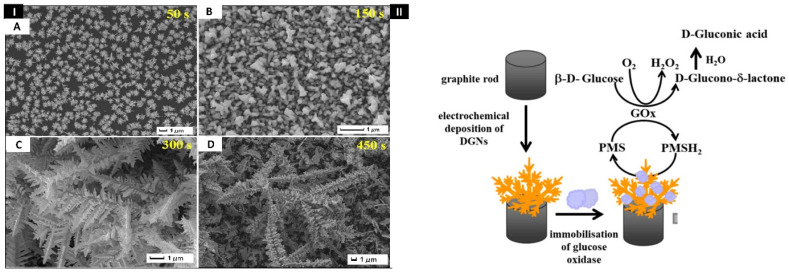
(**I**) Scanning electron microscopy (SEM) images of Ag−AuNDs sensors’ surface at electrodeposition time of (**A**) 50 s, (**B**) 150 s, (**C**) 300 s, and (**D**) 450 s, respectively. Reprinted with permission from [101]. Copyright 2020, Sun et al. (**II**) Pictorial representations show the electrodeposition of dendritic gold nanostructures (DGNs) and immobilization of glucose oxidase (GOx) on a graphite rod (GR) surface for recognition of glucose. Reprinted with permission from [102]. Copyright 2021, Ramanaviciene et al.

The high concentration of toxic chemicals in edibles poses major health hazards, making food contamination a serious concern. Melamine (2,4,6-triamino-1,3,5-triazine), a triazine compound, is highly poisonous and causes foodborne illnesses, namely renal failure, kidney stones, and even death. Likewise, the thiram molecules present in food are toxic to the humans’ mucosal membranes and skin, causing vomiting, nausea, and diarrhea. Sun et al. in 2020 have designed silver nanoparticles (AgNPs) decorated gold nanodendrites (AuNDs) biosensors for detecting melamine and thiram in food products quantitatively (Figure 3I). In this work, a template-free electrochemical deposition was used to create high-purity 3D AuNDs without the pollution of organic chemicals. The gold nanodendrites were created after optimizing the synthesis conditions with −1.5 V potential and a 300 s deposition time. Further, AuNDs surfaces were fabricated with citrate-reduced AgNPs to produce AgNPs coated AuNDs (Ag–AuNDs), which act as a hybrid SERS-active substrate. Because of the electromagnetic (EM) field enhancement, the synthesized composites displayed an outstanding Raman enhancement effect. The calibration plot showed the linearity between 0.01 and 5 mg/L, and 0.12 to 24 mg/L for melamine and thiram, respectively. The LODs for thiram and melamine were 86.1 μg/L and 7.38 μg/L, respectively. Real sample analyses have been carried out in apple juice and milk to detect thiram and melamine. In the future, the Ag–AuNDs substrate could serve as a potential platform to ensure food safety [101]. 

Diabetes is a serious public health issue that is rapidly spreading worldwide. Hence, developing a robust, reliable, rapid, accurate, and user-friendly device for diabetic patients is extremely important. Ramanaviciene et al. in 2021 [102] constructed a template-free biosensing platform for glucose detection, as shown in Figure 3II. This experiment used fine emery paper to polish the graphite rod (GR) electrode. Later, gold nanodendrites were electrochemically deposited using a solution containing HAuCl_4_ in potassium nitrate (KNO_3_) to form dendritic gold nanodendrites/graphite electrodes (DGNs/GR). DGN synthesis on the GR electrode was carried out using three electrochemical methods: pulse amperometry, constant potential amperometry (CPA), and DPV. The 6.0 mmol/L HAuCl_4_ concentration, 400 s time, and −0.4 V potential were optimum for electrodeposition. Glucose oxidase (GOx) solution was further immobilized on DGNs/GR to form (GOx/DGNs/GR). The as-prepared sensor showed a linear relationship between 0.1 and 9.97 mmol/L. The LOD was calculated as 0.059 mmol/L. Real sample analysis was done in human serum, and the developed device paved the way for future developments by employing other enzyme immobilization approaches and/or redox mediators [102]. 

Apart from this, we have included a comprehensive Table 1 that shows the nanodendrites-based sensors for recognizing different small molecules in real matrix, illustrating their sensing mechanisms, response time, readout systems, dynamic range, and LOD, etc.

## 6. Macro-Molecules Detection Using Metallic Nanodendrite-Based Sensors

Macromolecules are polymeric compounds with a high molecular mass and are made up of thousands of monomers. However, all macromolecules are not polymeric in nature. DNA, RNA, and proteins are the most common polymeric macromolecules, while lipid moieties and macrocycles are non-polymeric in nature. Macromolecules play a vital role as biomarkers, biocatalysts, and hormones in the biological system. This section has described different biosensors based on nanodendrites for macromolecule detection. 

**Figure 4:**
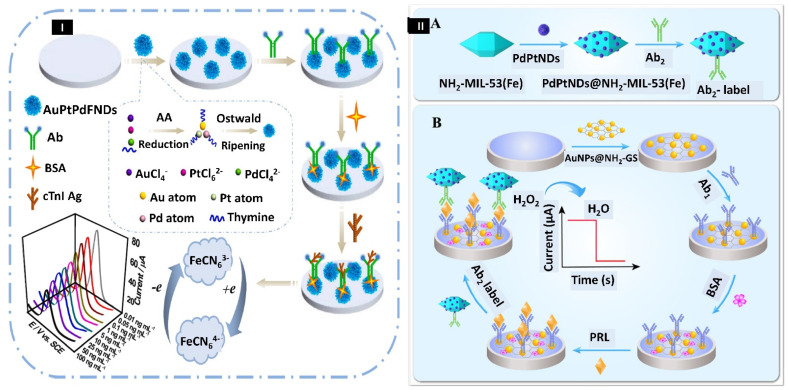
(**I**) Pictorial representation of AuPtPd trimetallic fluffy-like nanodendrites (FNDs)-based sensor for identification of cardiac troponin I (cTnI). Reprinted with permission from [126]. Copyright 2021, Cen et al. (**II**) Schematic showing the (**A**) Synthesis of Ab2-PdPt NDs@NH_2_-MIL-53(Fe), (**B**) Decoration of immunosensor. Reprinted with permission from [127]. Copyright 2021, Zhang et al.

Acute myocardial infarction (AMI), often known as a heart attack, is a fatal disorder that arises when blood circulation to the heart muscles is blocked suddenly, resulting in tissue damage. The mortality and morbidity rates of AMI are high, hence finding an accurate AMI diagnosis method is of utmost importance. The most prominent macromolecular biomarker for detecting myocardial damage and necrosis is cardiac troponin I (cTnI). Cen et al. 2021 have developed a highly selective and sensitive immunosensor with advanced nanomaterials, as displayed in Figure 4I. First, a one-pot thymine-mediated method was used to synthesize the porous trimetallic gold/platinum/palladium fluffy nanodendrites (AuPtPd FNDs). Then AuPtPd FNDs were dissolved in water and sonicated to get a uniformly dispersed suspension. The suspension mentioned above was coated on a GCE surface and dried. Further, the cTnI Ab solution was drop-coated on the AuPtPd FNDs customized GCE at 4 °C for 12 h, later bovine serum albumin (BSA) coating was applied to prevent non-specific binding. The immunosensor fabrication was characterized using CV and EIS, while the concentration dependent study of biomarkers was done by DPV. The calibration plot showed a wide linear range from 0.01–100.0 ng/mL and LOD of 3 pg/mL. The AuPtPd FNDs’ increased catalytic activity is primarily due to two factors: (i) the tri-metals’ synergistic activity accelerates electron transport, and (ii) the metallic dendritic nanostructures have a large surface area and many active sites, resulting in increased catalytic activity. In the near future, this biosensor can also be used to detect different cardiac biomarkers in real samples [126]. 

Prolactin (PRL), often called lactotrophin, is another proteinaceous macromolecule of 23 kDa. These 199 amino acids long hormone is secreted by the pituitary gland and performs many functions. It regulates lactation, reproduction, metabolism, the endocrine system, immunological system, and osmotic pressure. When there is an increase in prolactin levels, a condition called hyperprolactinemia occurs, which is linked with various diseased conditions, including primary hypothyroidism, prolactinoma, and polycystic ovary syndrome (PCOS). Whereas when there is a low level of PRL (<5 ng/mL) in the body, a greater incidence of metabolic disorders, namely glucose intolerance and insulin resistance, occurs. As a result, type 2 diabetes is more likely to occur. Zhang et al., 2021, have constructed a highly sensitive sandwich electrochemical biosensor to detect prolactin by utilizing a metal–organic framework (MOF) (Figure 4II). PdPt NDs were first produced at an ambient temperature via a one-step aqueous-phase method. Then, they were conjugated with MOF, NH_2_-MIL-53(Fe), followed by labelling with antibody 2 (Ab 2) to form Ab2-PdPt NDs@NH_2_-MIL-53(Fe). Further, gold nanoparticles (AuNPs) decorated with amino-functionalized graphene sheets (AuNPs@NH_2_-GS) were formed and fabricated on GCE and served as a matrix for faster electron transfer and immobilization of antibody 1 (Ab 1). Different concentrations of prolactin were incubated on the modified electrode. After that, the electrode surface was decorated with Ab2-PdPt NDs@NH_2_-MIL-53(Fe). The nanocomposite formed boosted the electrocatalytic activity for hydrogen peroxide (H_2_O_2_) reduction and increased the antibody immobilization by forming a bond between PdPt NDs and NH_2_ of Ab2. The electrochemical characterization was done by CV and EIS, while the concentration-dependent study was done by the amperometric i-t method. The calibration curve displayed the linearity between 0.001–500.0 ng/mL, and the LOD was estimated as 1.15 pg/mL. Hence, the suggested immunosensor has higher specificity, sensitivity, and better stability, implying that it has a great clinical diagnostic potential [127].

**Figure 5:**
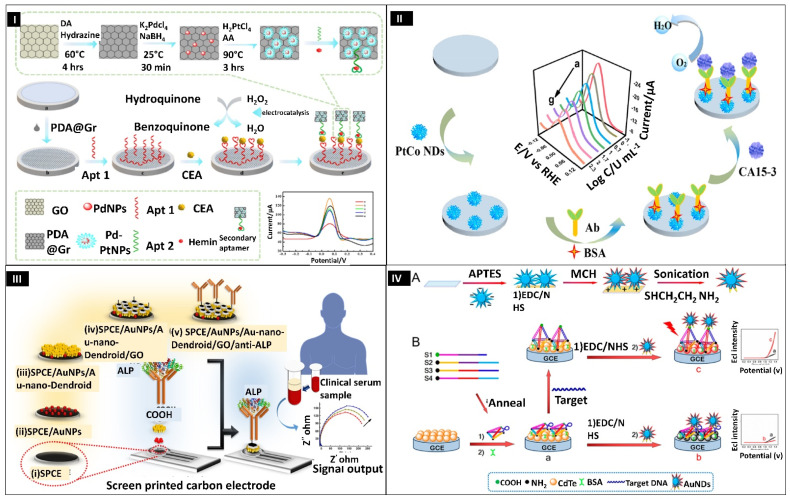
(**I**) Pictorial representation of the fabrication of aptasensor for detection of carcinoembryonic antigen. Reprinted with permission from [128]. Copyright 2021, Zhang et al. (**II**) Schematic representing the immunosensing of carbohydrate antigen 15-3 by bimetallic nanodendrites PtCo. Reprinted with permission from [129]. Copyright 2021, Ge et al. (**III**) Illustration depicting the preparation of SPCE/AuNPs/Au-nanodendroids/GO/anti-ALP sensor for detection of alkaline phosphatase (ALP). Reprinted with permission from [95]. Copyright 2020, Mahato et al. (**IV**) (**A**) Schematic denoting the step-by-step modification of surface, (**B**) Illustration showing the gold nanodendrites-based electrochemiluminescence sensor for nucleic acid detection. Reprinted with permission from [130]. Copyright 2018, Li et al.

Carcinoembryonic antigen (CEA) is a glycoprotein biomarker linked to various tumors, including lung, pancreatic, and colon cancers. As a result, quick and sensitive detection of CEA is crucial for clinical diagnosis and timely therapy. Zhang et al. 2021, used a conducting polymer polydopamine and bimetallic nanodendrites to develop an electrochemical aptasensor for CEA measurement (Figure 5I). First, polydopamine@graphene oxide (PDA@Gr) was prepared and fabricated on GCE, followed by the addition of aptamer 1 (Apt1) to form Apt1/PDA@Gr. The modified electrode was dipped in a solution of CEA at 4 °C for 40 min. Further Pd–Pt nanodendrites were formed on the surface to form PDA@Gr/Pd–PtNDs. This surface was used as a matrix for hemin/G-quadruplex (G4) immobilization, having a peroxidase-like activity, to produce the secondary aptamer. The secondary aptamer (Apt2) was trapped on the top of the electrode surface by the bonding of CEA and Apt2. A sandwich reaction occurred between the corresponding aptamers and CEA, whereby the Apt2 facilitated the hydroquinone (HQ) oxidation with H_2_O_2_. The signal was amplified and was proportional to CEA concentrations. The linear dynamic range is between 50 pg/mL–1.0 µg/mL, with a LOD of 6.3 pg/mL. PDA@Gr/Pd-PtNDs and hemin/G4 showed synergistic catalysis toward HQ, resulting in a dual amplification and increased sensitivity [128]. 

Similarly, carbohydrate biomarkers also serve a similar function as proteins and glycoproteins in disease diagnosis. Breast cancer, a malignant tumor in women, poses a significant health danger with a high fatality rate. One of the most well-known markers for breast cancer diagnosis is carbohydrate antigen 15-3 (CA15-3). Ge et al. 2021 have devised a label-free immunosensor using a directing growth agent, as shown in Figure 5II. In this experiment, the first alloyed nanodendrites of platinum and cobalt (PtCo NDs) were synthesized using a solvothermal process. Herein, L-carnosine was used as the co-directing agent for the formation of nanodendrites. Then, the PtCo NDs suspension was fabricated on GCE. The CA15-3 antibody solution was attached through Pt–N bonds on the PtCo NDs surface, followed by BSA binding. This unique dendritic architecture provides a high surface area and a greater catalytic active site for the ORR. A label-free surface for sensitive recognition of CA15-3 was built, based on ORR signal amplifications. The stepwise assembly of the biosensor was studied by DPV and EIS techniques, while DPV did the concentration-dependent study. The as-prepared sensor displayed a dynamic range between 0.1–200 U/mL with a LOD value of 0.0114 U/mL. This research paves the way for detecting other tumor biomarkers in clinical diagnosis [129].

Enzyme biocatalysts speed up the chemical reactions and, hence, metabolism. Alkaline phosphatase (ALP) is a phosphate-cleaving enzyme in various biofluids such as serum, saliva, and other secretions of the body. It plays a vital role in various physiological processes in the body. However, when it exceeds 350 U/L, it causes liver and bone disorders, hepatitis C, and cancer. Mahato et al. 2019 have designed an impedimetric immunosensor by using a new type of Au-branched structures (Au nanodendroids) (Figure 5III). First, AuNPs were electrochemically deposited on bare SPCE, followed by electrochemical deposition of Au nanodendroids. Further, graphene oxide sheets were coated on the sensor surface, and the surface was then activated for anti-ALP immobilization. The stepwise assembly of the biosensor was evaluated by LSV and EIS methods, while a concentration-dependent study by EIS. The linear range between 100 and 1000 U/L and LOD value of 9.10 (±0.12) U/L were achieved for ALP detection. The sensor developed is label-free, robust, and is easy to fabricate, as well as it is employed in point-of-care testing of ALP [95].

Nucleic acid, another macromolecule that acts as a blueprint for life, stores and expresses genetic information. For the sensing of the same, Li et al. constructed an electrochemiluminescence (ECL) emission sensor by utilizing a DNA tetrahedral scaffold as a switch to modulate the gap between cadmium–tellurium nanocrystals (CdTe NCs) and AuNDs (Figure 5IV). AuNDs, on the other hand, served as an ECL quencher and enhancer. In this experiment, GCE was first to be electrodeposited with a chitosan solution, followed by coating of CdTe NCs to form CdTe NCs/GCE. The further electrode was dipped in a DNA tetrahedron solution to attach DNA tetrahedron in a stem loop hairpin structure on the surface of the electrode. In turn off mode, the DNA tetrahedron was in a relaxed state on the CdTe NCs layer and the hairpin structure was closed. Due to Förster resonance energy transfer (FRET), the CdTe NCs ECL emission was suppressed by AuNDs, present on the top of the DNA tetrahedron. In the presence of target DNA, the hairpin structure gets converted to a rod-like form, and it increases the distance between CdTe NCs and AuNDs, leading to a considerable rise in ECL, generated by LSPR of AuNDs. EIS was used to characterize the surface of the electrode and the calibration plot showed the linear range from 1.0 to 500 fM and LOD of 30 fM. The ECL biosensor based on nanodendrites showed excellent sensitivity and selectivity in the serum sample; hence, we hope that it will open up new possibilities for the detection of other biomarkers with high sensitivity [130].

Apart from this, we have included a comprehensive Table 2 that shows the nanodendrites-based sensors for recognizing different small molecules in real matrix, illustrating their sensing mechanisms, response time, readout systems, dynamic range, and LOD, etc.

## 7. Detection of Cells Using Metallic Nanodendrite-Based Sensors

**Figure 6:**
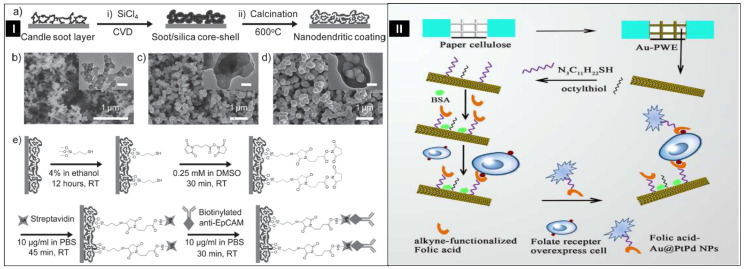
(**I**) (**a**) The schematic drawing shows the fabrication procedure of nanodendrite coating; (**b**) Images of a layer of candle soot captured using SEM and transmission electron microscopy (TEM) (insets); (**c**) Images of the soot/silica core–shell nanostructure by SEM and TEM (insets); (**d**) Images of the nanodendrites coat taken by SEM and TEM (insets) when candle soot was removed by calcination of the soot/silica core–shell nanostructure; (**e**) Illustration showing the fabrication of anti-EpCAM on nanodendrite coating for capturing cancerous cells. Reprinted with permission from [144]. Copyright 2013, Yang et al. (**II**) Pictorial illustration of folic acid decorated trimetallic nanodendrites Au@PtPd on paper for electrochemical identification of cancer cells. Reprinted with permission from [145]. Copyright 2009, Mani et al.

Nanodendritic-based biosensors also play a vital role in the sensing of harmful cells. However, very limited work has been done to detect whole cells. Two reported results are discussed here. In a fascinating study by Yang et al. in 2013, an underwater transparent nanodendritic silica coating-based device was designed for capturing cancer cells (Figure 6I). First, a three-step template method was used to fabricate nanodendrites. Further, biotinylated epithelial cell adhesion molecule antibody (anti-EpCAM) was immobilized to the nanodendritic coating. The modified coatings showed excellent efficiency of cell capture, by enhancing the topographic communications between the unique nanodendritic structure and cancer cells. The MCF 7 breast cancer cell line overexpresses a membrane antigen called the epithelial cell adhesion molecule, EpCAM. As a result, covalently adsorbed biotinylated anti-EpCAM on the surface captured and detected the cancerous cell. To investigate the therapeutic properties of the anti-EpCAM-fabricated nanodendrites, they conducted a number of tests to identify cancer cells in blood samples. MCF 7 cells, labelled with red dye, were injected into rat whole blood at different doses of 20, 50, 100, and 250 cells/mL. It resulted in a significant capture of 43–60% of spiking cells from blood under ideal circumstances. Hence, fabricated nanodendritic silica coating showed a dual-capability of effectively trapping and monitoring cancer cells [144]. 

Later, Ge et al. in 2015 have designed an electrochemical sandwich sensor by combining three metals to form Au@PtPd dendrites as displayed in Figure 6II. A simple approach was used to make Au@PtPd core–shell trimetallic dendritic NPs (Au@PdPt NPs), having a core of Au and shell of Pt−Pd alloy dendrites. First, in the sample zone of the paper working electrode (PWE), a porous layer was created by growing the AuNPs layer on the surface of cellulose fibers. Then, folic acid was decorated on its surface, and target cells were trapped on folic-acid-terminated Au-PWE. Further, the co-deposition of dendritic PtPd shells occurs on citrate-capped gold nanoparticles. The dendritic gold@platinum palladium nanoparticles (Au@PtPd NPs) displayed peroxidase-like activity and improved catalytic properties. Click chemistry was used to conjugate folic acid on dendritic Au@PtPd NPs’ surface, which further interacted with overexpressed folate receptors on the cancer cells’ surfaces. The detection of K-562 leukemic cells was performed using DPV curves, and the current was linear between 1.0 × 10^2^ and 2.0 × 10^7^ cells/mL. The LOD for cell concentration was estimated to be 31 cells/mL. Hence, the postulated robust non-enzymatic nanodendritic sensor with high sensitivity could be a promising choice for point-of-care diagnostics compared to enzymatic sensors. Enzyme-based sensors have some limitations, including low stability due to denaturation, as well as their preparation and purification being time-consuming and costly [145]. 

## 8. Conclusions and Future Prospects

Metallic nanodendrites have made remarkable progress in recent years and hold great promise in several biomolecular analyses. The first section highlighted the unique properties, various synthetic methods, and growth mechanisms of the branched metallic nanostructure. Different synthetic methods include GRR, seed-mediated, co-reduction, sonochemical reduction, laser-assisted methods, electroless deposition, and electrochemical deposition. In contrast to other methods of nanostructure synthesis, electrodeposition is very quick, requires no chemical reductants or oxidants, uses very little energy and raw materials, and is eco-friendly and cost-effective. In this review, we mainly focused on electrodeposited nanodendrites for detection of molecules due to the major advantages of this technology over other traditional methods. Several monometallic, bimetallic, and trimetallic nanodendrites based on Ni, Cu, Au, Pt, Ag, Co, and other metals have been utilized for diverse applications. The surface engineering of bimetallic and trimetallic nanodendrites to make varied dendritic composites is gaining wide interest because it tunes the nanodendrites’ electronic structure, boosts conductivity, increases the efficacy of the sensing layers, and reduces fouling effects. Different positively charged metal ions flow towards the negatively charged working electrode and get deposited on the surface in accordance with Faraday’s law, resulting in the formation of layers of nanodendrites. It is also worth mentioning that all these processes happen on the surface of electrodes/chips based on the electrochemical nanotuning optimization approach. The formation of these structures on different types of electrodes (shape, area, etc.) are optimized in every experimental setup. The nanodendrites on the electrode’s surface modify the electrode’s geometric/effective area, which leads to an accumulation of more charge carriers and thus increases the output current values. We have extensively discussed several biosensing strategies, including immunosensing and sandwich-type electrochemical sensing, based on branched metallic dendrites to detect small molecules, macromolecules, and cells. Label-free immunosensors provide potential applications for biomarkers screening as it shows sensitive detection with a wide linear range and low detection limit. In addition, label-free sensors reduce cost and sensing time, and their simplicity facilitates the formation of portable devices. These nanodendrite-based sensors exhibit excellent repeatability, even between five to ten analyses for various analytes, and have a standard deviation of less than 4%. Although, the features of 3D bimetallic nanodendritic materials are of great interest in developing sensors, there are only a few reports available. Hence, additional research is needed in this field to fabricate more efficient dendrites-based nanocomposites. More emphasis should be given to build miniaturized biosensing platforms that are robust and sensitive. We hope that the interesting features of metallic nanodendrites may open the doors in the field of nanomedicine in upcoming years. Additionally, the larger surface area and more adsorption sites of this unique 3D architecture can provide a surface for attaching a specific ligand, making it potentially beneficial for future diagnostic applications and the in vivo delivery of pharmaceuticals.

## Figures and Tables

**Table 1:** Various metallic nanodendrites-based biosensors for sensing of small molecules. (NR—not reported).

Sr. No.	Sensing Molecule	Detection Techniques	Description	Deposition Potential	Response Time	Real Sample	LDR	LOD	Reference
1	Uric acid	Amperometry	After being fabricated by ordered mesoporous carbon (OMC), screen-printed carbon electrode (SPCE) was electrodeposited with a three-dimensional (3D) dendritic nanomaterial of the palladium−platinum (Pd−Pt) alloy	0.14 V	NR	Serum	0.00025–0.80 mM	0.25 μM	[103]
2	**Pesticides**								
	(a) Paraoxon	DPV	First, BiVO_4_ was hydrothermally synthesized and characterized. It was then decorated on the screen-printed electrode for sensing of Paraoxon, an organophosphorus pesticide	NR	NR	River water	0.199–1.96 μM	0.03 μM	[104]
	(b) Dimethoate	Optical	Ag nanodendrite structures were developed on the optical fibers’ surface by a cost-effective laser-assisted photochemical method	NR	NR	NR	0.005–4 ppm	0.002 ppm	[105]
	(c) Permethrin	Optical	The procedure was initiated with the synthesis of SERS-active optical fiber substrates. Then, using a laser-assisted photochemical technique, silver (Ag) nanodendrites were deposited on the tip of the fiber core	NR	NR	NR	0.1–20 ppm	0.0035 ppm	[106]
3	**Metal ions**								
	(a) Selenium	Anodic stripping voltammetry	Glassy carbon electrode (GCE) modified with reduced graphene oxide (rGO) and further AuNDs was electrodeposited to form GCE/P-rGO/AuNDs	−0.2 V	NR	Seawater	3–300 nM	0.9 nM	[107]
	(b) Cadmium ion (Cd^2+^) and Lead ion (Pb^2+^)	DPASV	Bismuth nanodendrites (BiNDs) were fabricated by one-step electrodeposition of bismuth (Bi) and simultaneously detected Cd^2+^ and Pb^2+^ ions. Bromide ion (Br-) was used as a co-reagent to inhibit agglomeration of Bi	−2.8–−2.6 V	NR	Pure water, seawater, lake water	2–270 ppb	0.09 ppb (Cd^2+^) 0.05 ppb (Pb^2+^)	[11]
	(c) Mercury ion (Hg^2+^)	Electro chemiluminescent immunoassay	GCE was modified with gold nanoparticles (GNP20), and further nanodendritic structure of Pt/Pd was loaded on it. In this experiment, GNP50 was employed as a biocarrier to load more Pt/Pd NDs	NR	NR	Tap water, Lake water	0.05–1000 ng/mL	16 pg/mL	[108]
	(d) Hg (II), Cu (II), and Pb (II)	DPASV	One-step electrodeposition was used to create AuNDs structures on graphite pencil lead (GPL)	−0.3 V	NR	Lake water	1–50 ppb	0.18 ppb for Hg (II), 0.19 ppb for Cu (II), 0.12 ppb for Pb (II)	[109]
	(e) Nitrite	Amperometry	GCE modified with poly dimethyl diallyl ammonium chloride-reduced graphene oxide (PDDA-RGO), and further copper nanodendrites (CuNDs) were electrodeposited on it to form PDDA-RGO/ Cu NDs/GCE	−1 V	3 s	NR	1–15,000 μM	0.06 μM	[110]
4	Bisphenol A	CV, DPV	GCE modified with cetyl trimethyl ammonium bromide (CTAB), and further AuND were electrostatically deposited	NR	5 min	Drinking water	0.025–10 μM	22 nM	[111]
5	(a) Glucose	LSV	Cu nanodendrite foams (CuND foams) were electrodeposited on gold array electrodes under acidic conditions at negative overpotentials	−5.0 V	NR	Human serum	0.01–22.55 mM	NR	[112]
	(b) Glucose	Amperometry	A simple and easy displacement process, without any surfactants, was used to construct silver nanodendrites on copper rods	NR	<3 s	NR	0.02–7.4 mM	0.1 µM	[113]
	(c) Glucose	Amperometry	A simple electrochemical deposition approach was used to produce Ag nanodendrites on a Cu mesh substrate, which showed high electrocatalytic activity and SERS sensitivity	1.7 V	NR	Human urine	0.5–5 mM	0.005 mM	[114]
	(d) Glucose	CV	A Cu–Co alloy nanodendritic surfaces, with a hierarchical structure, was electrochemically prepared for detection of glucose	−0.80 V	5 s	Human blood	0.5 µM–14.0 mM	0.10µM	[10]
	(e) Glucose	Amperometry	Indium tin oxide (ITO) electrode was decorated with zinc oxide nanorods (ZnONRs) and further platinum nanodendrites (PtNDs) were synthesized on it via the chemical reduction method	NR	NR	Human blood	0.05–1 mM	0.03 mM	[115]
6	Cholera toxin	DPV	Using poly-(2-cyano-ethyl)pyrrole (PCEPy), dendritic gold architecture was functionalized with antibodies. Here, conductive polypyrrole polymer PCEPy and directed electrochemical nanowire assembly (DENA) were combined to facilitate functionalization.	NR	NR	NR	NR	1 ng/mL	[116]
7	(a) H_2_O_2_	Chronoamperometry	GCE modified with p-benzoic acid-2,2′:5′,2″-terthiophene (TBA) polymer and further gold nickel (AuNi) dendrites were deposited electrochemically to detect H_2_O_2_	–0.8 V	3 s	Cancer cell, normal cell	5–40 nM, 80 nM–30μM, 200 μM–2.5 mM	5 nM	[117]
	(b) H_2_O_2_	Amperometry	A simple and easy displacement process, without any surfactants, was used to construct silver nanodendrites on copper rods	NR	<3 s	NR	0.2–19.2 mM	0.1 µM	[113]
	(c) H_2_O_2_	CV	GCE electrode modified with (Pd/Pt-NDs) and rGO, which was coated with poly (diallyldimethylammonium chloride) (PDDA)	0.018 V	5 s	Fetal bovine serum (FBS)	0.005–0.5 mM	0.027 μM	[118]
	(d) H_2_O_2_	CV	A copper–cobalt (Cu–Co) alloy nanodendritic surface, with a hierarchical structure, was electrochemically prepared for detection of glucose	−0.80 V	5 s	Human blood	1.0 μM–11 mM	0.75 μM	[10]
	(e) H_2_O_2_	CV	DPV technique was used to electrodeposit bismuth nanodendrites (BiNDs) on gallium nitride (GaN) electrode	−0.05 V	NR	Fetal bovine serum (FBS), milk, tap water	10 μM–1 mM, 1–10 mM	5 μM	[119]
8	Pyrazinamide (PZA) drug	DPV	GCE was altered with zinc–aluminum layered double hydroxide (Zn–Al LDH), and further nanodendritic silver (AgNDs) were electrodeposited on the surface	–0.3 V	NR	Human serum and urine	9.0 × 10^−7^–5.2 × 10^−4^ mol/L	7.2 × 10^−7^ mol/L	[120]
9	**Amino acids**								
	(a) Tryptophan	DPV	GCE was modified by new polymeric materials made from oligolactides by cross-linking with tetracarboxylated thiacalix [4] arene in a cone, partial cone and 1,3-alternate configurations and then silver was deposited by potential cycling in the polymer film pores	0.7 V	NR	Sedative medicine	0.1–100 µM	0.03 µM	[121]
	(b) Tryptophan	SWV	First, Ag dendrites were synthesized, and then polythiophene (PT)–Ag nanodendrites composite were formed by electrostatic interaction and fabricated on the GCE surface	NR	NR	Soybeans extract	200 nM–400 μM	20 nM	[122]
10	Acetaminophen	Amperometry	First, PDDA-coated gold dendrite, and poly (sodium 4-styrenesulfonate) (PSS) coated rGO was synthesized. Finally, rGO-gold dendritic surface was constructed by self-assembly of both for acetaminophen detection	NR	NR	Tablets, human urine	0.07–3000 μM	0.005 μM	[123]
11	Hydrazine	Amperometry	ITO electrodes were modified with silver dendritic structures by using an aqueous solution of AgNO_3_ and KNO_3_ without any surfactants	−0.80 V	<5 s	Tap water, distilled water, and river water samples	100–1700 μM	0.5 μM	[124]
12	Paracetamol	CV, Chronoamperometry	Silver nanodendrites and its composite with graphene oxide (GO) were constructed by galvanic replacement method and dropcasted on GCE	NR	<3 s	NR	0.5–10 mM	0.025 μM	[125]

**Table 2:** Various metallic nanodendrites-based biosensors for sensing of macromolecules. (NR—not reported).

Sr. No	Sensing Molecule	Detection Techniques	Description	Deposition Potential	Response Time	Real Sample	LDR	LOD	Reference
1	**Proteins**								
	(a) Human epididymis protein 4	DPV	Trimetallic AgPtCo nanodendrites were synthesized by convenient one-pot method	NR	NR	HE-4-positive ovarian cancer patients	0.001–50 ng/mL	0.487 pg/mL	[131]
	(b) Alpha-fetoprotein (AFP)	Amperometry	Graphene (NH2-GS) doped mesoporous Au@Pt nanodendrites (NH2-GS/Au@Pt) and poly-dopamine coated N-doped multi-walled carbon nanotube (PDA-N-MWCNT) was used to synthesize sandwich electrochemical immunosensor for AFP sensing	NR	NR	NR	0.1 pg/mL–10 ng/mL	0.05 pg/mL	[132]
	(c) Alpha-fetoprotein (AFP)	CV	First, poly (diallyldimethylammonium chloride) decorated molybdenum disulfide nanosheet (MoS2) was synthesized and hybridized with polypyrrole nanotubes. Then, platinum nanodendrites were fabricated to form Pt NDs/PDDA/MoS2@PPy NTs	NR	NR	Human serum	50 fg/mL–50 ng/mL	17 fg/mL	[133]
	(d) Ovalbumin (OVA)	Optical	Antibody-modified silver dendrites were coupled with surface-enhancedRaman scattering (SERS) phenomena for identification of OVA	NR	30 min	Milk	NR	5 μg/mL	[134]
2	Hemoglobin	DPV	On the Au electrode surface, haemoglobin (Hb)-imprinted poly(ionic liquids) (HIPILs) were built to create Au/AuND/HIPILs. Gold nanodendrites were earlier used to alter the Au electrode surface	−0.9 V	NR	Bovine blood sample	1.0 × 10^−14^ –1.0 × 10^−4^ mg/mL	5.22 × 10^−15^ mg/mL	[135]
3	**Nucleic acid**								
	(a) DNA	Chronoamperometry	The one-pot method was utilized to construct PdPt nanodendrites, which acted as a carrier for the DNA probe. Further, the PdPt NDs were combined with melamine	NR	NR	Human serum	1 fmol/L–1 nmol/L	0.33 fmol/L	[136]
	(b) miRNA	DPV	Nanodendritic gold structure was electrodeposited on the ITO/Ti/Au, and further graphene was deposited on the surface.	−1.8 V	NR	NR	0.43 pM–1.13 nM	0.34 nM	[137]
	(c) lncRNAs	CV	Graphene oxide/Au/horseradish peroxidase surface was decorated with Pt–Pd bimetallic nanodendrites to form PtPd/BND/BNF@GO/Au/HRP nanocomposites. Thionine or a detecting probe was coated over Au particles	NR	NR	Serum	1.00 × 10^−3^–1.00 × 10^3^ pM/mL	0.247 fM/mL	[138]
4	Enolase	DPV	GCE modified with AuNPs and further forms GCE/Au/Ab1/BSA/NSE surface. Finally, TB/WP6@PdPt-Ab2 were deposited on the surface	NR	NR	Human serum	0.0003–100.00 ng/mL	0.095 pg/mL	[139]
	**Carbohydrates**								
	(a) Carbohydrate antigen 15-3 (CA15-3)	DPV	First, Au@Pt core–shell nanodendritic crystals (Au@Pt NCs) were synthesized by one-pot wet-chemical strategy. Then, it was dispersed homogenously with ferrocene-grafted-chitosan (Fcg-CS) on GCE surface	NR	NR	Serum	0.5–200 U/mL	0.17 U/mL	[140]
5	(b) Carbohydrate antigen 15-3 (CA15-3)	DPV	Using a one-pot solvothermal technique, and co-structure-directing agent, L-carnosine platinum-cobalt nanodendritic (Pt-Co NDs) surfaces were made	NR	NR	Human serum	0.1–200 U/mL	0.0114 U/mL	[129]
6	Insulin hormone	Amperometry	Antibody 1 was immobilized on glassy carbon electrode (GCE) surface altered with gold nanoparticles (AuNPs). Finally, antibody 2 conjugated Pt-Co-Cu nanodendrites were electrodeposited	−0.2 V	NR	Serum	0.2–2000 pM	0.08 pM	[141]
7	17 β-estradiol (E2) hormone	EIS	Boron doped diamond (BDD) electrode surface was used to grow dendritic gold by a double template method. Further 17 β estradiol (E2) aptamers were functionalized on the surface of the Au/BDD electrode by covalent bonding (Au-S) to capture E2.	NR	NR	Water	1.0 × 10^−14^ to 1.0 × 10^−9^ mol/L	5.0 × 10^−15^ mol/L	[142]
8	**Glycoprotein** Carcinoembryonic antigen (CEA)	DPV	Bimetallic core–shell rhodium@palladium nanodendrites (Rh@Pd NDs) synthesized on MWCNT, functionalized with sulfo group (MWCNTs-SO_3_H) to prepare Rh@PdNDs/MWCNTs-SO_3_H composite surfaces	NR	NR	Human serum	25 fg/mL to 100 ng/mL	8.3 fg/mL	[143]

## Data Availability

Not applicable.
